# Evaluating the Need for Enhanced Alzheimer's Disease Education: A Cross‐Country Analysis of Public Awareness and Symptoms Prompting Medical Consultation

**DOI:** 10.1002/alz70858_103777

**Published:** 2025-12-25

**Authors:** Hannah Brown, Georgia Giannakopoulou, Sandra Teoh, Nazira Jainis, Hasifullah Ibrahim, Amber King, Joseph I Vindici, Simone Gabriele

**Affiliations:** ^1^ Ipsos, London, London, United Kingdom; ^2^ Ipsos, Kuala Lumpur, Kuala Lumpur, Malaysia; ^3^ Ipsos, Kuala Lumpur, Kuala Lumpur, United Kingdom; ^4^ Ipsos, New York, NY, USA

## Abstract

**Background:**

Early recognition of Alzheimer's Disease (AD) symptoms is crucial for timely diagnosis. Yet, limited public awareness of symptoms may hinder early detection. In order to assess the need for more widespread education, this study evaluated which symptoms are likely to prompt individuals to visit a doctor and whether they feel adequately informed about AD.

**Method:**

An online survey was conducted in August 2024 with 804 adults aged 18 years or older from the US (*n* = 106), EU4+UK (France, Germany, Italy, Spain, UK, *n* = 517), Japan (*n* = 106), and China (*n* = 75). Respondents were screened to ensure some understanding of, but not currently suffering from, AD. Without indicating that the list comprised common mild cognitive impairment (MCI) symptoms, participants were asked to select up to three symptoms that would prompt them to seek medical help. They were also asked whether they believe enough information is shared about AD.

**Results:**

“Memory loss that disrupts day‐to‐day life” was the most commonly cited symptom triggering an individual to seek medical help (60% overall), highest in the US (63%) and lowest in Japan (47%). Other symptoms were notably selected less frequently. “Decreased or poor judgment” was among the least commonly chosen (15%), but was selected more often in Japan (37%) and China (36%) than in the US (10%) or EU4+UK (9%). Respondents aged 70+ (*n* = 109) were more likely to be prompted by “Forgetting the word you wanted to use” (35%) than those aged 18–35 (18%, *n* = 212). 66% of all respondents believed not enough information is shared about AD, with this perception strongest in EU4+UK (72%) versus US (54%) and China (45%).

**Conclusions:**

In this study cohort, memory loss was most likely to prompt medical consultation. However, other common symptoms of MCI were less frequently cited, indicating potential gaps in awareness of the broader spectrum of symptoms associated with AD. Furthermore, the majority perception that insufficient information about AD is shared underscores the need for enhanced public education initiatives. Improving awareness of diverse symptoms could facilitate earlier AD diagnosis and better patient outcomes.

